# Progressive modulation of the human olfactory bulb transcriptome during Alzheimer´s disease evolution: novel insights into the olfactory signaling across proteinopathies

**DOI:** 10.18632/oncotarget.18193

**Published:** 2017-05-23

**Authors:** Mercedes Lachen-Montes, María Victoria Zelaya, Víctor Segura, Joaquín Fernández-Irigoyen, Enrique Santamaría

**Affiliations:** ^1^ Clinical Neuroproteomics Group, Navarrabiomed, Departamento de Salud, Universidad Pública de Navarra, Pamplona, Spain; ^2^ IDISNA, Navarra Institute for Health Research, Pamplona, Spain; ^3^ Pathological Anatomy Department, Navarra Hospital Complex, Pamplona, Spain; ^4^ Bioinformatics Unit, Center for Applied Medical Research, University of Navarra, Pamplona, Spain; ^5^ Proteored-ISCIII, Proteomics Unit, Navarrabiomed, Departamento de Salud, Universidad Pública de Navarra, Pamplona, Spain

**Keywords:** Alzheimer, neurodegeneration, dementia, olfactory bulb, transcriptomics

## Abstract

Alzheimer´s disease (AD) is characterized by progressive dementia, initially presenting olfactory dysfunction. Despite the olfactory bulb (OB) is the first central structure of the olfactory pathway, we lack a complete molecular characterization of the transcriptional events that occurs in this olfactory area during AD progression. To address this gap in knowledge, we have assessed the genome-wide expression in postmortem OBs from subjects with varying degree of AD pathology. A stage-dependent deregulation of specific pathways was observed, revealing transmembrane transport, and neuroinflammation as part of the functional modules that are disrupted across AD grading. Potential drivers of neurodegeneration predicted by network-driven transcriptomics were monitored across different types of dementia, including progressive supranuclear palsy (PSP), mixed dementia, and frontotemporal lobar degeneration (FTLD). Epidermal growth factor receptor (EGFR) expression was significantly increased in the OB of AD and mixed dementia subjects. Moreover, a significant increment in the activation of signal transducer and activator of transcription 3 (STAT3) was exclusively detected in advanced AD stages, whereas total STAT3 levels were specifically overexpressed in mixed dementia. Furthermore, transcription factors deregulated in the OB of mixed dementia subjects such as cAMP Responsive Element Binding Protein 1 (CREB1) and AP-1 Transcription Factor Subunit (c-Jun) were not differentially modulated at olfactory level across AD grading. On the other hand, olfactory expression of this signal transducer panel was unchanged in PSP and FTLD subjects. Taken together, this study unveils cross-disease similarities and differences for specific signal transducers, providing mechanistic clues to the intriguing divergence of AD pathology across proteinopathies.

## INTRODUCTION

Although olfactory involvement may also appear in healthy non-demented elderly subjects [1], olfactory dysfunction is present in up to 90% of AD patients [2]. Some studies suggest that olfactory dysfunction is an early event of AD, preceding the appearance of typical AD symptoms, such as memory loss, and dementia. The olfactory bulb (OB) is the first central structure of the olfactory pathway in the brain [3]. An OB atrophy and a significant reduction in olfactory performance have been detected in AD respect to control subjects using MRI and PET technologies [4, 5]. From a neuropathological point of view, olfactory centres are involved in early Braak stages [6], and OB pathology correlates with cortical AD pathology [7–9]. In view of these data, an in depth biochemical characterization of the neurodegeneration that occurs in the OB is mandatory as a first step for understanding early smell impairment in AD. Although neuroanatomical, volumetric, and histological approaches have been the gold standard techniques employed to characterize the OB functionality, little attention has been focused specifically on the molecular composition of the OB from the perspective of high throughput molecular technologies [10, 11]. Different transcriptomic studies have been attempted to discover novel regulatory mechanisms associated with AD pathogenesis in brain areas differentially affected by the disease [12]. Nevertheless, no study to date has addressed whether specific patterns of gene expression is associated to the development of human AD-related pathology at olfactory level in a stage-dependent manner. We consider that deciphering the progressive transcriptome-wide alterations that occurs in the OB derived from human AD cases with different Braak staging, might help develop early diagnosis and identify potential therapeutic targets for AD. In this study, we have analyzed the progressive modulation of the OB transcriptome across neuropathological stages of AD, in order to increase our knowledge about the pathophysiological mechanisms that are disturbed during the AD neurodegeneration in the OB. 249 differential genes were detected between controls and AD-related phenotypes, pinpointing specific pathways, gene interaction networks, and potential novel therapeutic targets that are modulated in specific AD stages. Interestingly, the OB transcriptome exploration in parallel with a cross-disease analysis including different proteinopathies, has revealed distinct modulation of specific signal transducers, providing new avenues of research into the role of olfactory signaling across different types of dementias.

## RESULTS AND DISCUSSION

**Figure 1:**
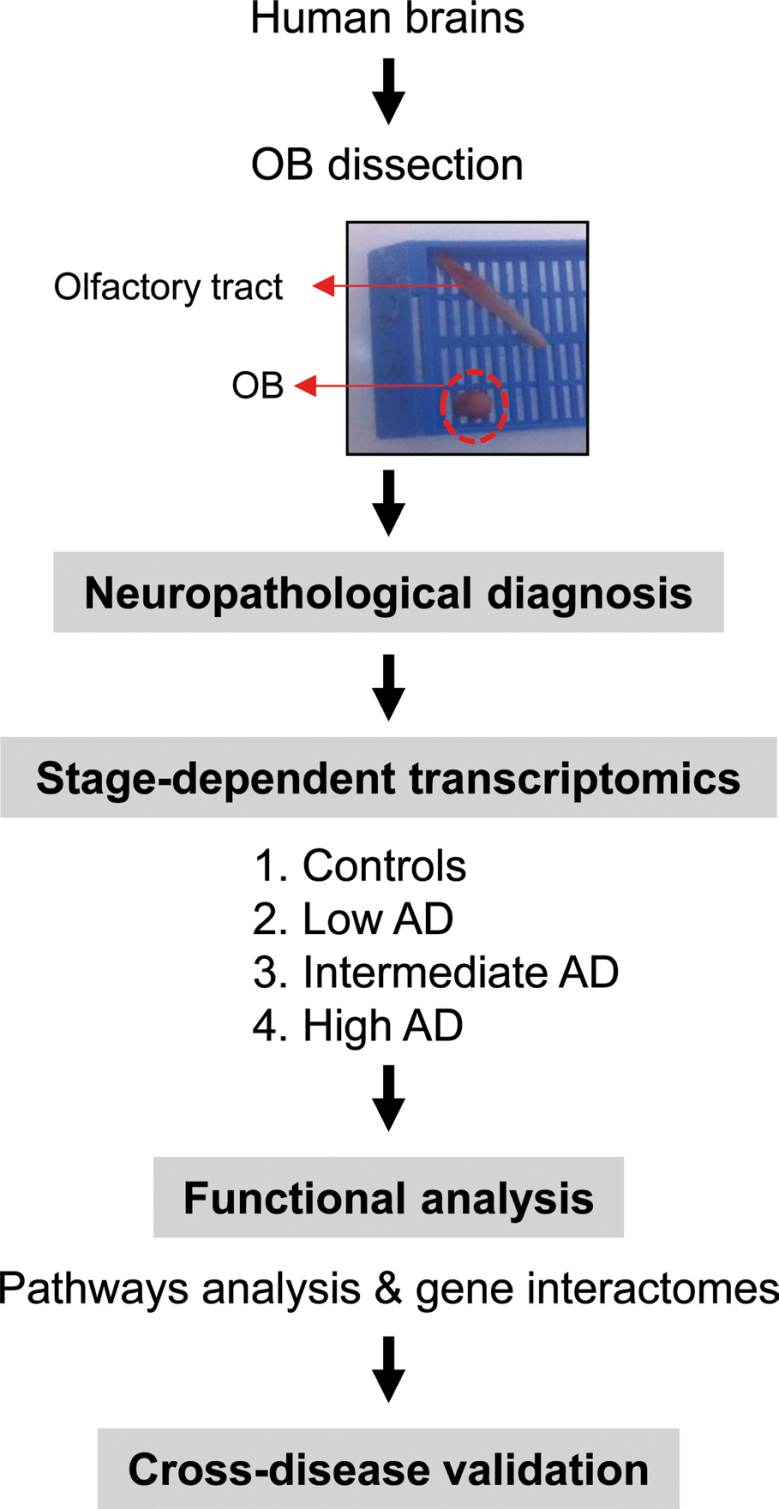
An overview of the workflow used for the characterization of **OB** transcriptome during AD evolution

During the last decade, gene expression profiling of postmortem tissue has greatly increased our knowledge about the pathophysiological mechanisms that occur in affected brain structures during AD progression [12]. With the aim to identify downstream aberrant gene expression related to beta-amyloid and Tau deposits across AD phenotypes, the temporal lobe–hippocampus and the frontal–prefrontal cortex has been the most studied areas [12–16]. However, loss of smell is involved in early stages of AD, partially due to an imbalance in the OB functionality [17]. Albeit olfactory impairment is considered an important clinical marker and predictor of AD progression [18], the mechanisms governing this dysfunction are still poorly understood. Transcriptome profiling has revealed multiple metabolic alterations in the OB of a rat AD model [19], however, the progression of the disease in rodent models does not correlate well with human AD [20], being necessary genome-wide studies in human olfactory tissue with neuropathologically well-defined AD-associated changes (Figure 1).

### OB transcriptome dynamics during AD progression

**Figure 2:**
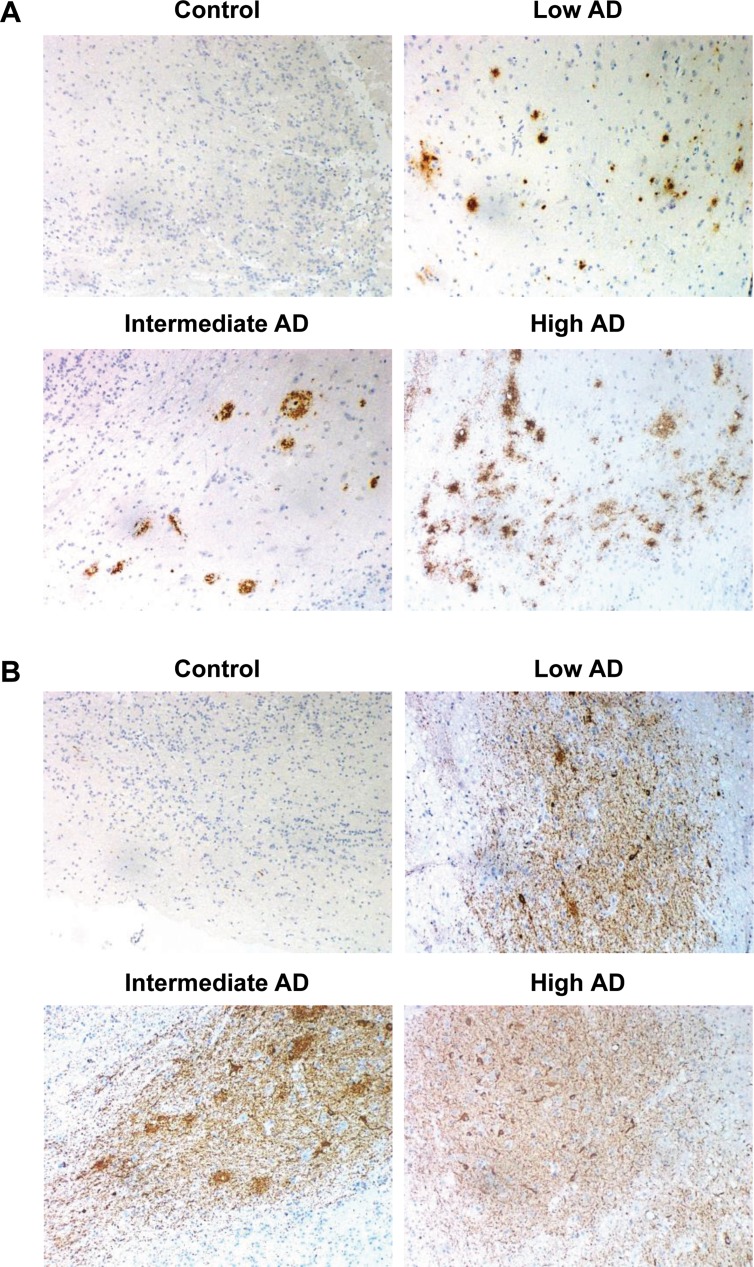
Representative immunohistochemical analysis of β-amyloid and phospho-Tau in the OB across AD stages (**A**) Control: negative staining of β-amyloid in the anterior olfactory nucleus (AON). Low AD: mild compact deposits of β-amyloid in the AON. Intermediate AD: sparse neuritic plaques of β-amyloid in the AON. High AD: mild neuritic and diffuse plaques of β-amyloid in the AON. (**B**) Control: isolated neuropil threads of phospho-Tau protein in the glomerular layer of the OB. Initial AD: Moderate neuropil threads and tangles of phospho-Tau protein. Intermediate AD: Severe deposit of neuropil threads of phospho-Tau protein. Advanced AD: Severe neuropil threads and tangles of phospho-Tau protein in the AON (All images are 20×).

**Figure 3:**
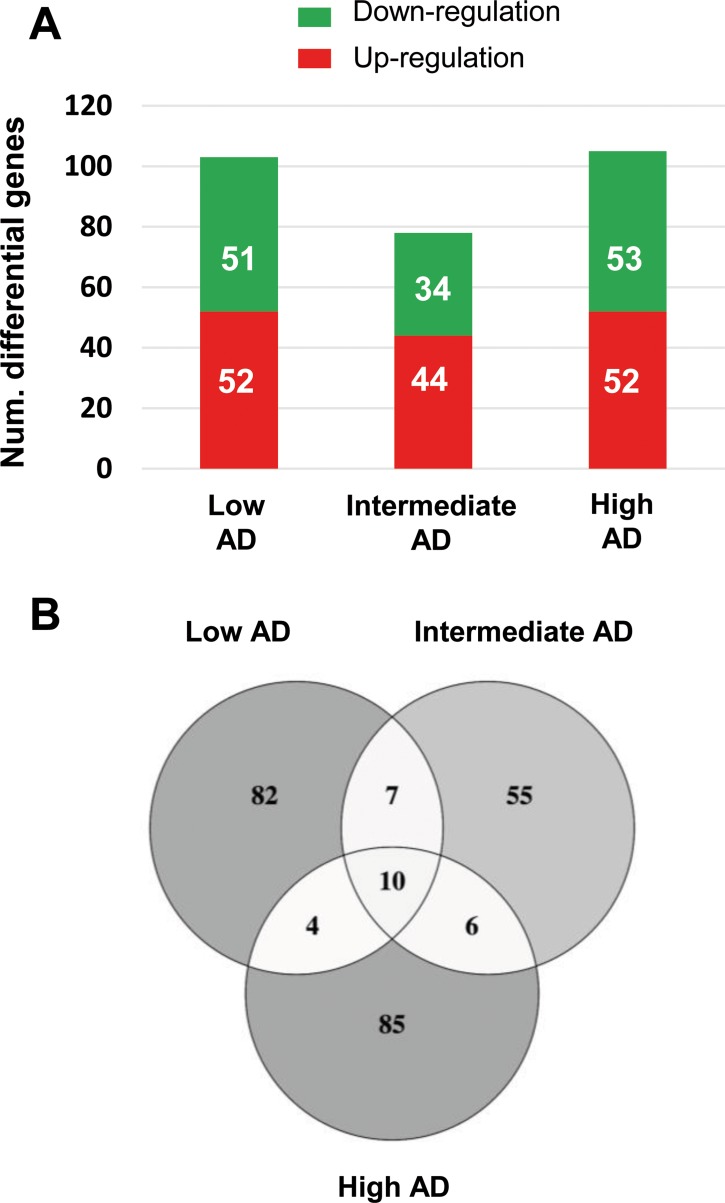
Differentially expressed genes in the OB across AD-related phenotypes (**A**) Differential olfactory transcriptome distribution across AD stages. (**B**) Venn diagram of common and unique differential genes between AD stages. The distribution of common and distinct protein-coding genes in low, intermediate, and high AD stages is shown.

The immunohistochemical study of β-amyloid and phospho-Tau protein in the cases included in this study (Table 1), allowed us to confirm the presence of neuropathological proteins in the OB of subjects with distinct stages of AD (Figure 2), confirming the involvement of OB in pre-clinical stages of the disease. To further understand the olfactory molecular background contributing to the progression of AD, we have performed a differential OB transcriptome analysis in order to detect early and stage-dependent molecular events underlying the progression of the disease at olfactory level (Figure 1). As shown in Figure 3, 103, 78, and 105 differentially expressed protein-coding genes were detected in initial, intermediate, and advanced AD stages respectively (Supplementary Table 2). In our sample set, the distribution between up-regulated and down-regulated protein coding-genes was very similar across AD grading (Figure 3A). As expected, we detected a substantial heterogeneity within the same Braak staging. This may be due to unpredictable confounders such as clinical, environmental, behavioral and agonal factors (i.e medication, substance abuse and health status prior to death) [21]. However, 10 genes were modulated across all stages (Figure 3B), suggesting a potential role during AD progression (Table 2), although we can not discard that this subset of olfactory genes may be also primary related to the neurodegenerative process that occur in other neurodegenerative diseases with common smell impairment. Some of these genes are involved in synaptic plasticity (*EGFLAM*), zinc transporter (*SLC39A11*), retinoid carrier (*LCN10*), and sodium and carbohydrate transport (*SLC5A11*). Other differential expressed genes overlapped between initial-intermediate stages, intermediate-advanced stages, and initial-advanced stages (Table 2). We have investigated whether the differential OB transcriptomic fingerprint has been partially reflected in previous transcriptomic studies performed in different brain structures across AD pathology [15, 22–27]. According to our integrative meta-analysis (Supplementary Table 3), most of the differential OB genes have not been previously proposed as differential molecular features in hippocampal structures affected by the disease, serving as a foundation for new areas of investigation into the role of olfactory signaling in human AD. However, due to the OB pathology correlates with cortical AD pathology [7–9], we compared our differential gene sets with the differential expressed genome that previously showed significant expression correlation to Braak stage and cerebral atrophy in prefrontal cortex from AD subjects [16]. Thirteen OB differential genes in initial stages (*KLC1, RAB7L1, C8orf46, GRM8, DCC, TMEM9, DDA1, HPCAL1, C15orf37, SYT13, VIP, RGS4, SST*), seven differential expressed OB genes in intermediate stages (*OR2T2, LOR, DCLRE1C, HMOX2, UBE2NL, SYT13*), and eight differential OB genes in advanced stages (*ZNF443, PHF17, CEP68, UIMC1, SMAD5, ELF1, PTPN2, CASP1*) present a significant expression correlation to Braak staging at cortical level [16]. Moreover, twenty OB early-deregulated genes (*C15orf37, C8orf46, DCC, DDA1, GIMAP7, GRM8, HPCAL1, KLC1, PDE10A, RASAL1, RGS4, SST,TMEM204, TMEM9, VIP, TMSB15B, RAB7L1, SYT13, EGFLAM, SLC39A11*), sixteen OB intermediate-affected genes (*TMSB15B, RAB7L1, SYT13, ESAM, EGFLAM, SLC39A11, CTXN3, DCLRE1C, GTF3C6, HLA-DRA, HMOX2, LOR, NBPF1, OR2T2, RASL11B, SNRPN*), and fifteen OB advanced-deregulated genes (*EGFLAM, SLC39A11, ESAM, CASP1, CD58, CEP68, CHRM4, ELF1, PHF17, PTPN2, SERPINH1, SMAD5, TUBA1A, UIMC1, ZNF443*) showed a good correlation with cerebral atrophy [16]. Although our stage-dependent analysis presents a limited number of study population, these data shed new light on the potential coordinated regulation of specific gene modules across AD-related brain structures, reinforcing the molecular correlation between OB and cortical AD pathology beyond the presence and distribution of beta-amyloid and phospho-Tau protein [7–9]. Using data mining-based methods for proteome-scale protein-protein interaction predictions [28], we have generated the potential interactome for human APP (β-amyloid precursor protein) and Tau protein (Supplementary Table 4), detecting some OB differentially expressed protein-coding genes as potential APP and/or Tau interactors. Specifically, differentially expressed genes in initial stages like *RASAL1*, *TUBB4A*, and *BTK* genes are potential APP interactors, whereas *MAPK8IP1*, and *HSPA1B* genes (deregulated in advanced stages) may be potential Tau interactors. Although these predictive assumptions should be experimentally validated, this information may be useful to generate new working hypothesis to clarify the relationship between both neuropathologic substrates in AD at olfactory level. *KLC1* gene (Kinesin 2) is a common interactor between both neuropathological substrates (Supplementary Table 4). Moreover, *RASAL1*, *TUBB4A*, and *KLC1* are also deregulated in cortical areas from AD patients [24, 27], being *KLC1* a modifier of the beta-amyloid accumulation [29]. Interestingly, kinesin 2 protein levels were significantly increased in the OB from initial and advanced AD stages (Supplementary Figure 1). In addition, *HSPA1B* gene (up-regulated in advanced AD at the level of OB) is also up-regulated at protein level in hippocampus from AD subjects [30]. *RASAL1* and *TUBB4A* genes (up-regulated in initial AD at the level of OB) are down-regulated in hippocampal proteome at all pathologic stages of AD [30]. All these evidences suggest that AD pathology modulates the gene/protein expression of most APP and Tau interactors mentioned in this study in a spatial and stage-dependent manner across AD brains.

**Table 1:** Summary of selected cases included in this study

		Duration PMI			Pathological diagnosis	IHQ:	βA in OB	IHQ: TAU in OB	
Cases	age	sex	(years)	(hours)	NIA-AA criteria	MP	DP	Tangles	neurites
Control									
C1	72	M		9	Thal 1 Cerad 1 no tau deposit	−	−	−	+
C2	103	M		3	No protein deposit+vascular disease	−	−	−	+
C3	81	F		3.3	PART (Braak I)+vascular disase	−	−	−	+
C4	61	M		8	PART (Braak I)	−	−	+	−
**Low AD**									
I1	88	M	1	3.45	AD (A2B1C2)	++	+	++	++
I2	85	F	8	2	AD (A2B1C1)	−	−	+	+
I3	80	M	5	3	AD (A2B1C1)	++	++	++	+++
I4	75	F	n.d	6	AD (A1B1C1)	−	−	+	+
I5	72	F	n.d	4	AD (A1B1C1)	−	−	+	+
**intermediate AD**									
M1	85	M	12	3.3	AD (A2B2C2)	−	+	+++	+++
M2	97	F	9	n.d	AD (A2B2C2)	n.d	n.d	n.d	n.d
M3	77	M	17	1.5	AD (A2B2C1)	−	−	++	++
M4	86	F	9	3	AD (A2B2C2)	−	+	++	++
**High AD**									
A1	77	F	16	4	AD (A2B3C3)	+	+++	+++	+++
A2	70	M	4	2.5	AD (A3B3C3)	++	+++	++	+++
A3	89	M	13	3	AD (A2B3C3)	+	+++	+++	+++
A4	86	M	8	2.5	AD (A3B3C3)	+	−	+	++
A5	93	M	3	2.4	AD (A3B3C3)	+	+++	+++	+++

The neuropathological assessment was performed according to Thal phases, adapted CERAD score, NIA-AA guidelines and PART criteria. Aβ immunopositivity was scored on a 4-tiered scale as: (−) negative, (+) 1–2isolated Aβ depositions, (++) 3–4Aβ depositions, and (+++) >4 Aβ depositions. Graduation of phospho-TAU deposit: (−) negative +: low; ++: intermediate; +++ high. PMI: post-mortem interval; n.d: not determined; MP: Mature plaques; DP: Diffuse plaques.

**Table 2:** Common differentially expressed genes across AD staging

Gene name	Gene description	LOW AD		INTERMEDIATE AD		HIGH AD	
		p-Val	FC	p-Val	FC	p-Val	FC
EGFLAM	EGF-like, fibronectin type III and laminin G domains	0.002	0.48	0.003	0.48	0.009	0.55
LOC391636	chromosome 9 open reading frame 78 pseudogene	0.000	0.55	0.003	0.62	0.000	0.57
RN5S344	RNA, 5S ribosomal 344	0.003	0.60	0.003	0.59	0.000	0.41
SCARNA2	small Cajal body-specific RNA 2	0.004	0.72	0.002	0.68	0.010	0.25
FLJ39739	uncharacterized FLJ39739	0.007	1.33	0.005	1.37	0.004	1.37
MRPL23-AS1	MRPL23 antisense RNA 1 (non-protein coding)	0.004	1.34	0.009	1.32	0.000	1.53
SLC39A11	solute carrier family 39 (metal ion transporter), member 11	0.002	1.54	0.001	1.70	0.000	1.98
LCN10	lipocalin 10	0.009	1.61	0.002	1.82	0.000	2.00
CCHCR1	coiled-coil alpha-helical rod protein 1	0.000	1.74	0.004	1.48	0.004	1.46
SLC5A11	solute carrier family 5 (sodium/glucose cotransporter), member 11	0.001	2.30	0.002	2.30	0.010	1.89
TMSB15B	thymosin beta 15B	0.006	0.72	0.006	0.71	n.s	n.s
SLIT3	slit homolog 3 (Drosophila)	0.005	1.46	0.008	1.45	n.s	n.s
RNU7-76P	RNA, U7 small nuclear 76 pseudogene	0.003	1.54	0.010	1.47	n.s	n.s
HCRTR1	hypocretin (orexin) receptor 1	0.003	1.55	0.007	1.51	n.s	n.s
TMEM186	transmembrane protein 186	0.006	1.55	0.008	1.55	n.s	n.s
RAB7L1	RAB7, member RAS oncogene family-like 1	0.003	1.58	0.007	1.52	n.s	n.s
SYT13	synaptotagmin XIII	0.002	1.74	0.010	1.57	n.s	n.s
SNORD116-27	small nucleolar RNA, C/D box 116-27	n.s	n.s	0.000	0.42	0.003	0.53
YTHDC1	YTH domain containing 1	n.s	n.s	0.008	0.56	0.006	0.56
TREX2	three prime repair exonuclease 2	n.s	n.s	0.002	0.59	0.002	0.60
TK2	thymidine kinase 2, mitochondrial	n.s	n.s	0.001	0.61	0.007	0.68
IP6K3	inositol hexakisphosphate kinase 3	n.s	n.s	0.004	1.40	0.004	1.38
ESAM	endothelial cell adhesion molecule	n.s	n.s	0.006	1.44	0.005	1.44
RRP7B	ribosomal RNA processing 7 homolog B (S. cerevisiae)	0.006	0.20	n.s	n.s	0.001	0.11
SNORA36B	small nucleolar RNA, H/ACA box 36B	0.001	0.36	n.s	n.s	0.001	0.38
ZNF45	zinc finger protein 45	0.009	0.73	n.s	n.s	0.004	0.70
PPP1R13L	protein phosphatase 1, regulatory subunit 13 like	0.006	1.34	n.s	n.s	0.002	1.40

Although the analysis of the OB transcriptome provides a unique window into their biochemistry and dysfunction across AD stages, there are potential limitations of our study that warrant discussion. We have processed all cellular layers present in the bulk OB, giving novel insight into the gene-expression in this olfactory area. However, the OB is composed by intermixed multiple cell types with intricate architecture and connectivity [31], and information about specific-cell types where mRNAs originated from is lost in our dataset. The implementation of novel workflows that allow the exploration of olfactory cell-type specific transcriptomes [32] would complement the output of our nonbiased profiling of the OB transcriptome, minimizing the effect of multiple neuronal microenvironments, and deciphering the specific role of each olfactory neuronal population during AD progression.

### Progressive modulation of olfactory pathways across AD staging

**Figure 4:**
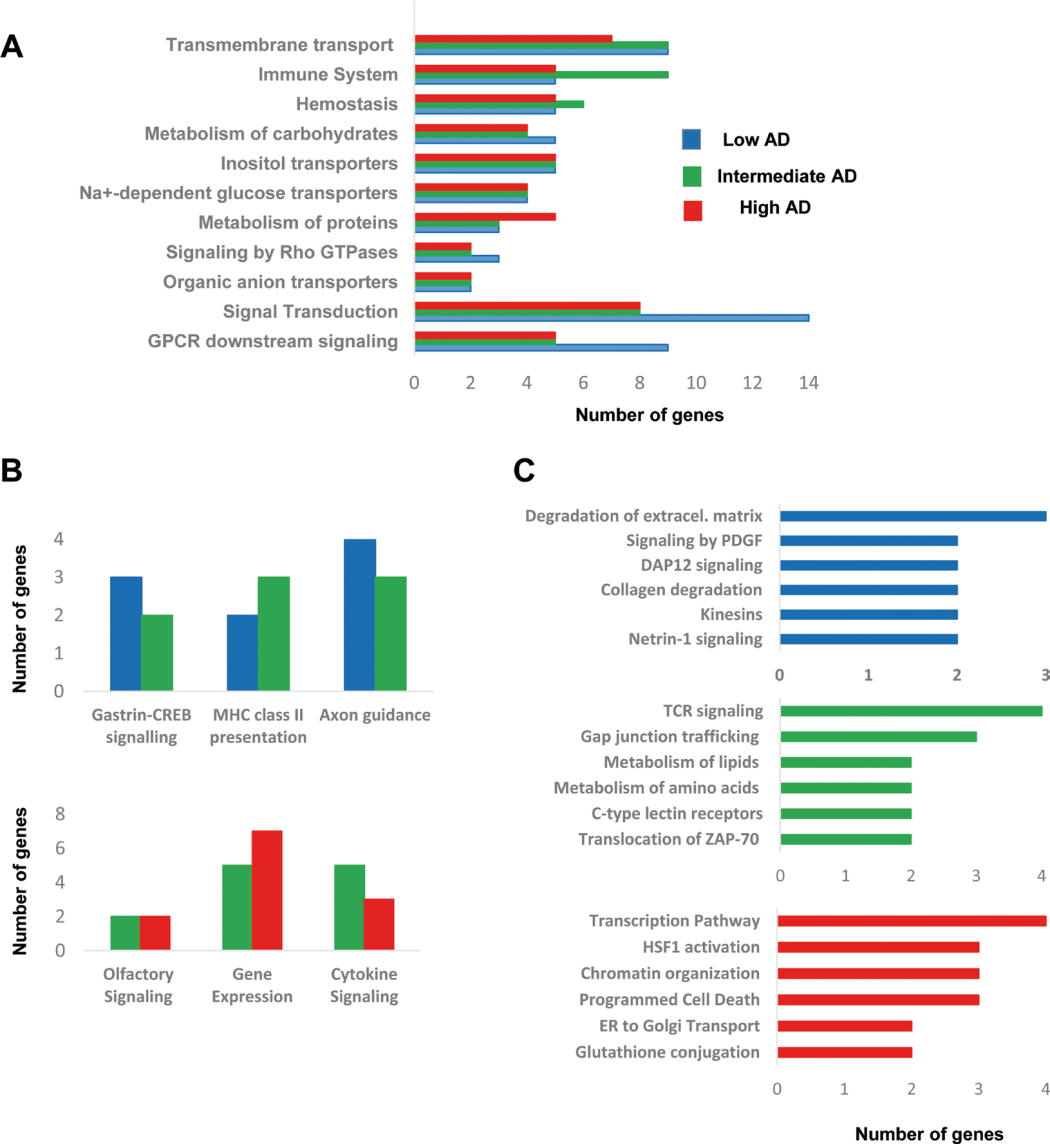
Functional metrics of the differential OB transcriptome across AD staging Specific pathway analysis for the differential OB transcriptome detected in each AD stage is shown. Blue, green, and red bars correspond to functional deregulated categories in low, intermediate, and high AD stages respectively.

To obtain a functional genomic perspective, differential transcriptomes were analyzed for higher-level organization of genes into common biological pathways using the Reactome database [33] (Supplementary Table 5). As shown in Figure 4, our results point out a stage-dependent deregulation of specific pathways. Gene clusters involved in hemostasis, metabolism of carbohydrates, and metabolism of proteins were mapped across AD stages (Figure 4A), confirming previous observations obtained at protein level using proteomic workflows [34]. Moreover, a de-regulation of genes involved in signal transduction, immune system, and molecular transport was also evidenced across AD staging (Figure 4A), reinforcing the idea that cellular signaling and neuroinflammation are common driving forces of AD pathology across brain structures [12]. Gastrin-CREB signaling is involved in neurogenesis and cognitive impairment at hippocampal level [35], suggesting that the slight alteration in this pathway in initial-intermediate stages (Figure 4B) might play a role in the disruption of olfactory neurogenesis that occur in AD [36]. In addition, OB *HLA-DR* genes involved in MHC class II presentation pathway were up-regulated in intermediate stages (Figure 4B), in accordance with previous transcriptomic experiments performed in cortical structures from AD patients [16, 24, 27]. In line with these findings, an increase in HLA-DR immunopositive microglia across all layers of the cortex has been detected in post-mortem AD brains [37]. A deregulation of sensory perception of smell has been proposed from transcriptomic information extracted from prefrontal cortex derived from AD subjects [16]. Accordingly, olfactory receptor (OR) gene dysregulation has been demonstrated in entorhinal and frontal cortex during AD progression [38]. In our case, we found several de-regulated OR genes during AD progression at the level of OB (Figure 4B). In particular, *OR5M1*, and *OR2T2* genes were down-regulated in intermediate stages, while *OR2T8*, and *OR6J1* genes were over-expressed in advanced stages (Supplementary Table 2). These data suggest that the presence of neuropathological substrates at the level of OB triggers a minor alteration in the OR transcriptome across AD stages, being necessary further developments that enable the analysis of OR family at protein level in the context of AD [11, 39, 40]. Moreover, a slight deregulation of a subset of functional categories was observed in specific AD stages. As shown in Figure 4C, degradation of extracellular matrix, signaling by PDGF, and DAP12 were specifically mapped in initial stages. In line with our observations, gene modules regulated by PDGF, and DAP12 (or TYROBP) are disrupted in cortical structures from AD subjects [16]. Specifically, TYROBP expression is restricted to cells involved in the innate immunity [41], and is one of the causal regulator of the activated immune system network in late-onset AD [16]. On the other hand, metabolism of lipids, and aminoacids together with TCR signaling by ZAP-70 were exclusively detected in intermediate stages (Figure 4C). In advanced stages, gene clusters related to transcription, HSF1 activation, and ER to Golgi transport were specifically deregulated (Figure 4C).

### Modulation of gene interactome networks in the OB across AD stages

**Figure 5:**
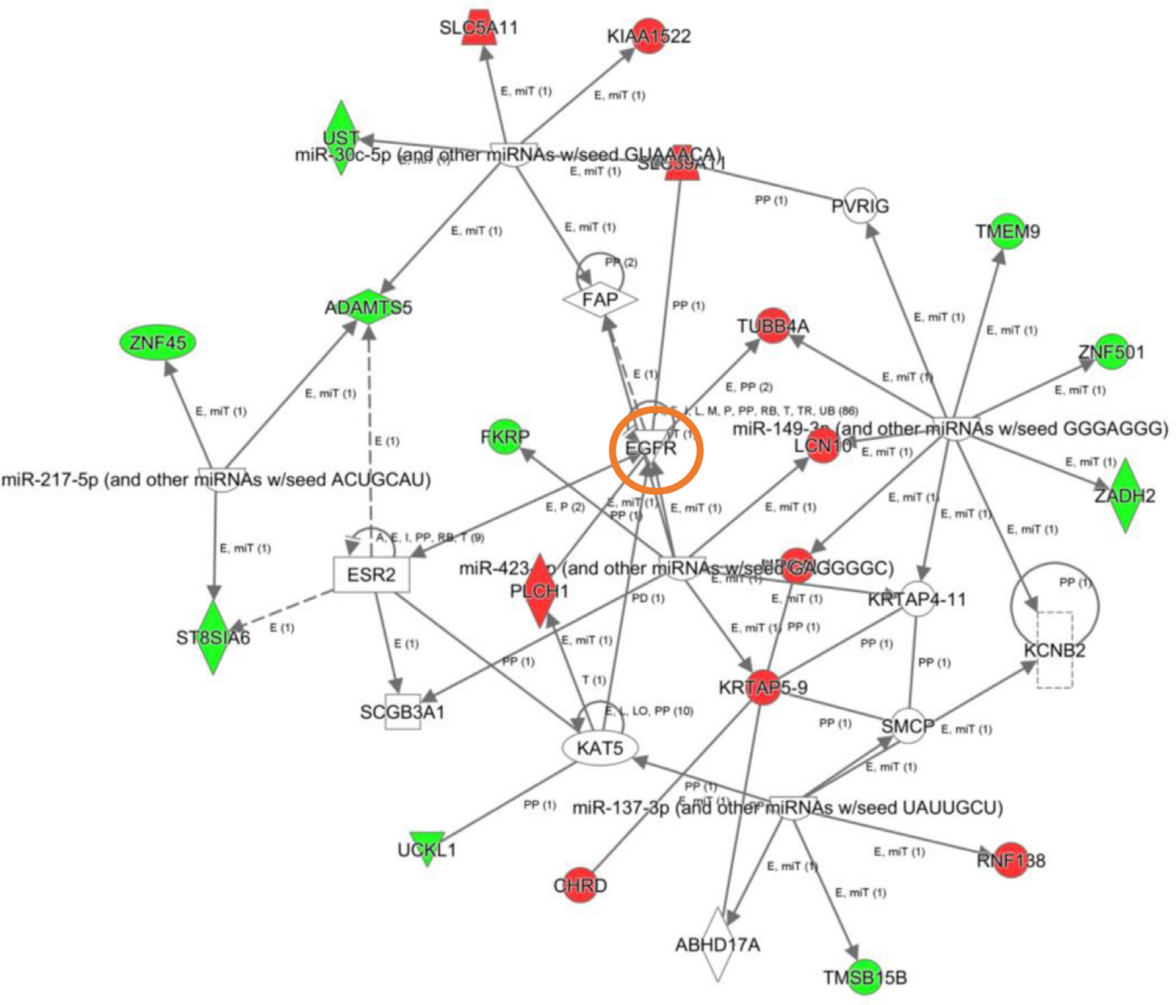
High-scoring gene interactome map for early differentially expressed genes in the OB during AD progression Visual representation of the relationships between differential expressed genes and functional interactors in low AD stage. Dysregulated genes are highlighted in red (up-regulated) and green (down-regulated). Continuous and discontinuous lines represent direct and indirect interactions respectively. The complete legend including main features, molecule shapes, and relationships is found at http://ingenuity.force.com/ipa/articles/Feature_Description/Legend.

**Figure 6:**
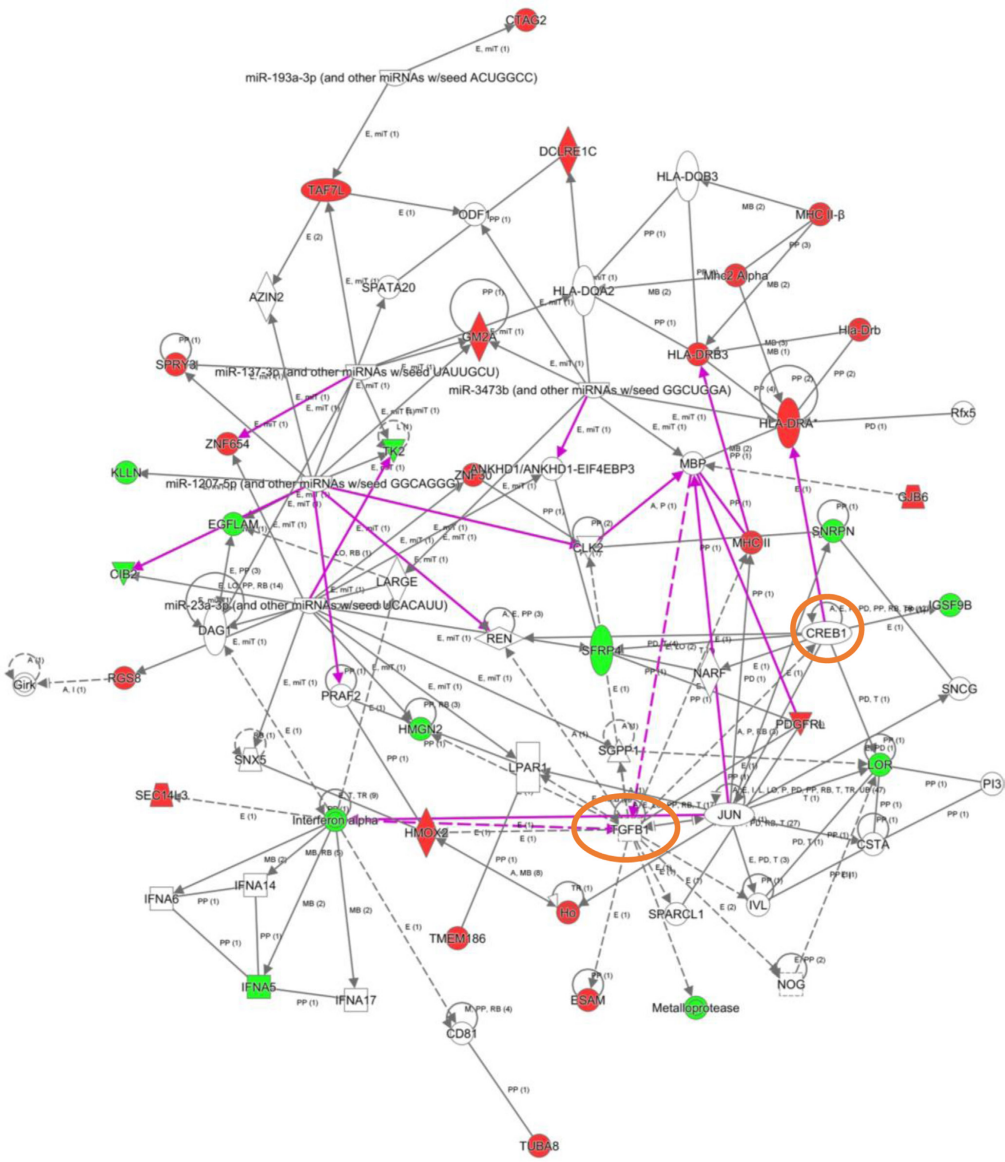
High-scoring gene interactome map for OB differentially expressed genes in intermediate AD stages Visual representation of the relationships between differential expressed genes and functional interactors in intermediate stages. In this case, two networks were merged to facilitate the global interpretation (purple lines correspond to novel functional links after merging). Dysregulated genes are highlighted in red (up-regulated) and green (down-regulated). Continuous and discontinuous lines represent direct and indirect interactions respectively. The complete legend including main features, molecule shapes, and relationships may be found at http://ingenuity.force.com/ipa/articles/Feature_Description/Legend.

**Figure 7:**
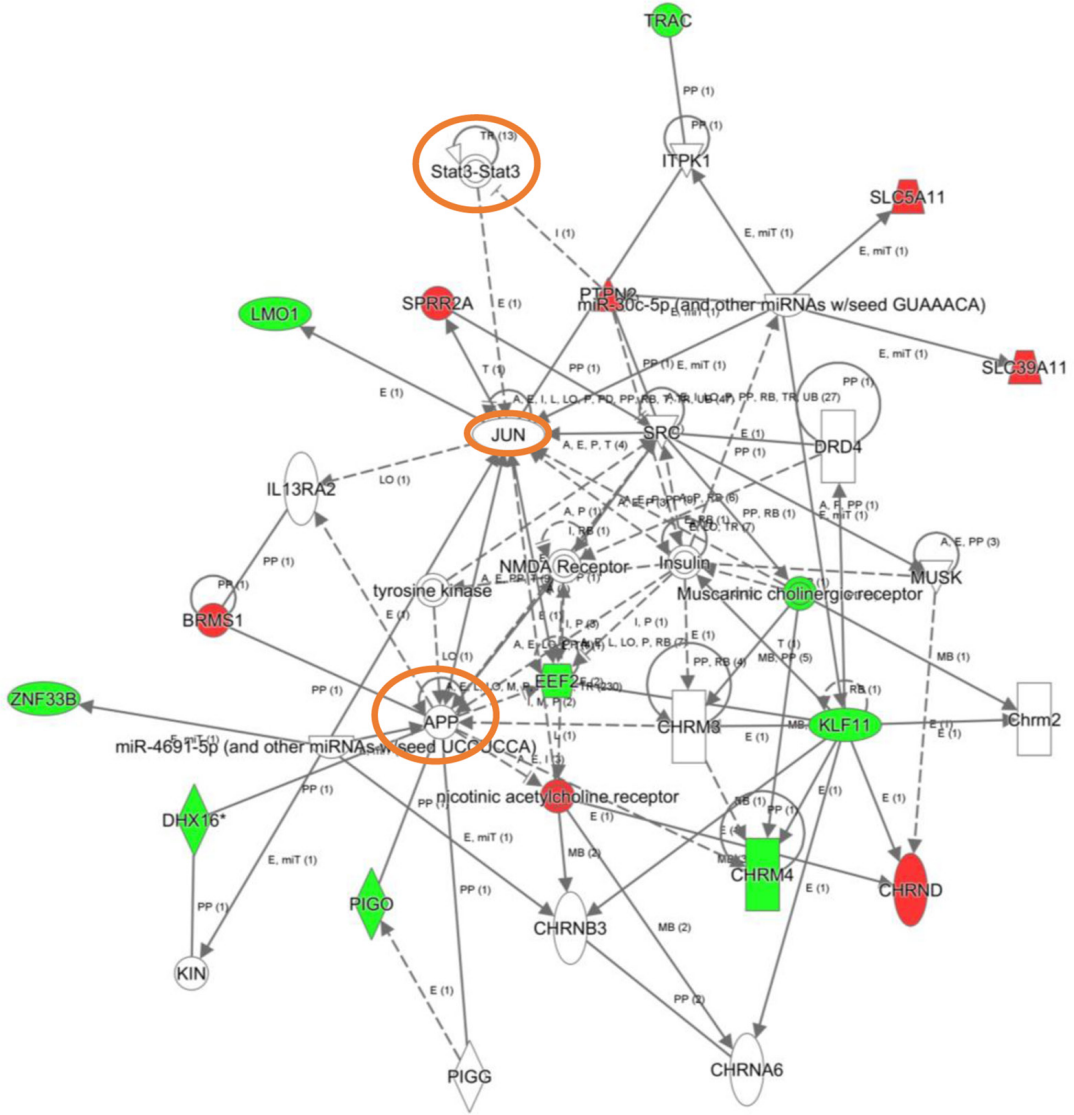
High-scoring gene interactome map for OB differentially expressed genes in high AD stages Visual representation of the relationships between differential expressed genes and functional interactors is shown. Dysregulated genes are highlighted in red (up-regulated) and green (down-regulated). Continuous and discontinuous lines represent direct and indirect interactions respectively. The complete legend including main features, molecule shapes, and relationships may be found at http://ingenuity.force.com/ipa/articles/Feature_Description/Legend.

To explore the cooperative action among differentially expressed genes, we performed gene-scale interaction networks merging the olfactory genes that tend to be de-regulated across AD staging. Using IPA software, a gene interactome map has been constructed for each AD stage (Figures 5–7). In this case, the integrative network-based approach allowed us to: i) elucidate the biological function and molecular context of the deregulated genes in each neuropathological stage, ii) establish a framework to map interaction between deregulated genes and network modules across AD grading, and iii) to define potential causal regulators of the stage-dependent networks that may be considered as gene targets to modulate the disease progression at olfactory level. In initial AD stages, the top deregulated pathways proposed by IPA were estrogen biosynthesis (*p*-value: 7,12E-03), cAMP-mediated signaling (*p*-value: 1,02E-02), and Gi Signaling (*p*-value: 1,15E-02), suggesting a central function of EGFR in the functional network (Figure 5). In intermediate stages, communication between innate and adaptive immune cells (*p*-value. 1,59E-03), and antigen presentation pathway (*p*-value: 5,02E-03) were the top deregulated pathways, being TGF-beta, and CREB1 potential nodes of the network (Figure 6). In advanced stages, the functional module composed by STAT3, c-Jun, and APP nodes appears as one of the main axis in the network (Figure 7).

### Protein expression of predictive interactome hubs across AD grading: Focus on olfactory EGFR, CREB1, TGF-beta, c-Jun and STAT3

**Figure 8:**
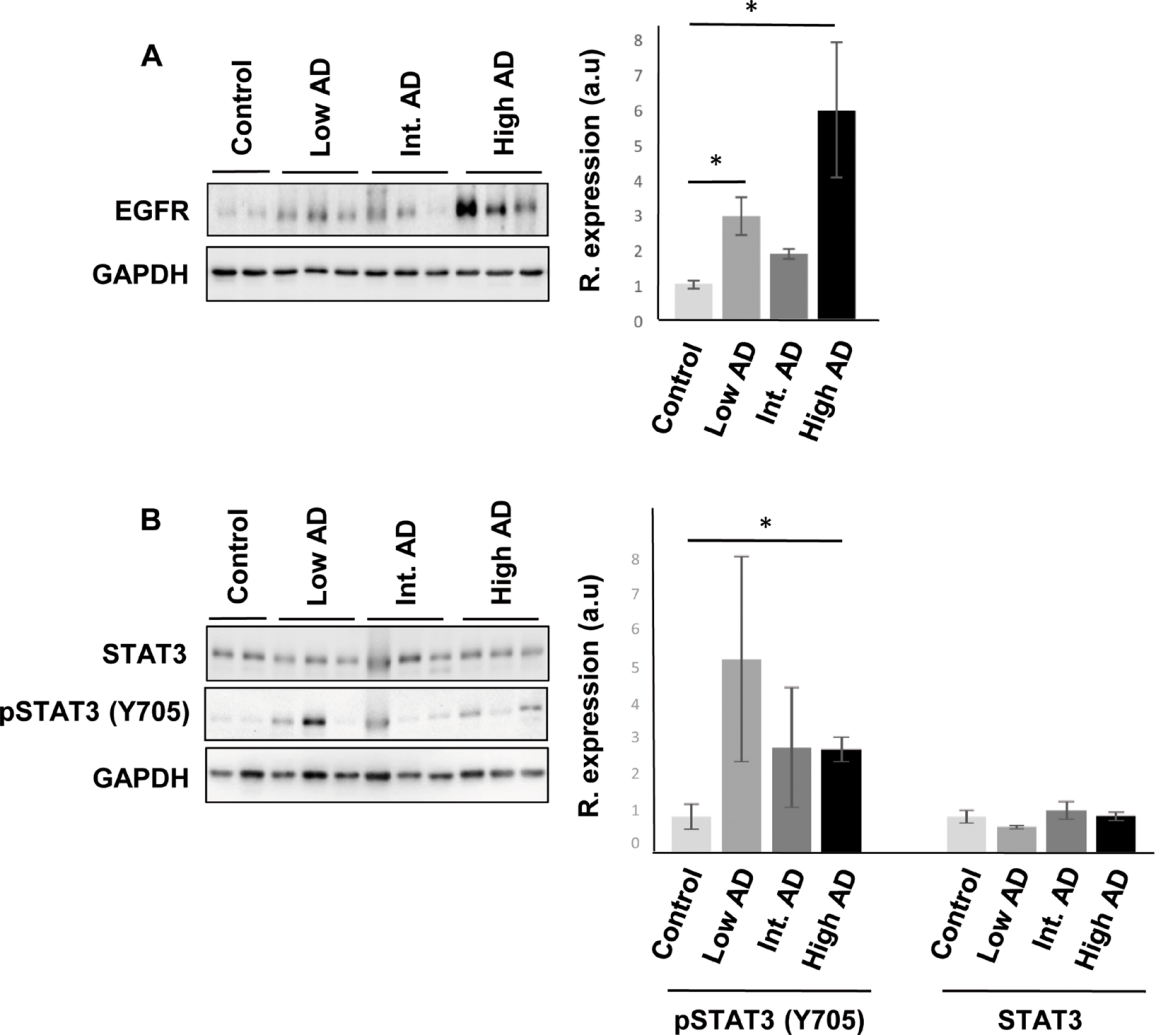
Olfactory bulb protein expression of EGFR, and STAT3 across AD staging (**A**) Representative Western blot gels to detect olfactory EGFR across AD grading. (**B**) Protein expression of Total STAT3, and active STAT3 (Y705) in the OB during AD progression. Right panels shows histograms of band densities. Data are presented as mean ± SEM from 3 independent OB samples per group. *P < 0.05 vs control group.

Even though changes in their expression were not detected in our transcriptomic workflow, the alteration of some of their targets may be compatible with a dysregulation of their functionality during AD progression at the level of OB. For that, subsequent experiments were performed in order to monitor the OB protein expression of these signal transducers across AD stages. Although a deficient EGFR signaling affects the OBs in mice, being necessary for olfactory learning, and discrimination [42–44], an increment in olfactory EGFR protein expression was significantly detected in initial and advanced AD stages (Figure 8). Interestingly, intense EGFR expression has been also observed in hippocampal and cortical neuritic plaques from patients with pathologically confirmed AD [45], suggesting that abnormal EGFR signaling could contribute to cognitive impairment in AD [46]. CREB1 is at a central converging point of activated pathways during the processes of synaptic strengthening and memory formation, and targeted therapeutic strategies focusing on augmentation of CREB-mediated transcription might prove beneficial for the enhancement of both processes in initial stages of AD [47–49]. Disruption of these mechanisms in AD results in a reduction of CREB1 activation with accompanying memory impairment [50, 51]. At olfactory level, most of the well-known activity-dependent CREB target genes such as *C-FOS*, *FOSB*, *BDNF*, *NR4A2*, and *EGR1* [50] were unchanged across AD stages (with the exception of *CYR61* that was up-regulated in advanced stages) (Supplementary Table 2). This transcriptomic fingerprint might partially corroborate the unmodified activation state of phosphorylated CREB (Ser133) observed at olfactory level during AD progression (Supplementary Figure 2). In relation to the predictive findings observed in Figure 7, it has been proposed that activated STAT3 is involved in the responsiveness of microglia to beta amyloid [52], being a common inducer of astrocyte reactivity in AD [53]. Moreover, STAT3 has been recently proposed as an upstream regulator in late onset AD at cortical level [27]. Accordingly, we observed an increment in the phosphorylation state of STAT3 (Y705) in advanced AD stages (Figure 8). In accordance with previous studies, the late STAT3 activation observed in the OB suggests an impairment in the differentiation process of olfactory neurons in advanced stages of the disease [54]. On the other hand, other hubs proposed by the interaction network analysis such as TGF-beta and c-Jun presented unmodified protein levels across AD grading (Supplementary Figure 2).

### Protein expression of olfactory EGFR, CREB1, TGF-beta, c-Jun and STAT3 across Alzheimer-related co-pathologies

**Figure 9:**
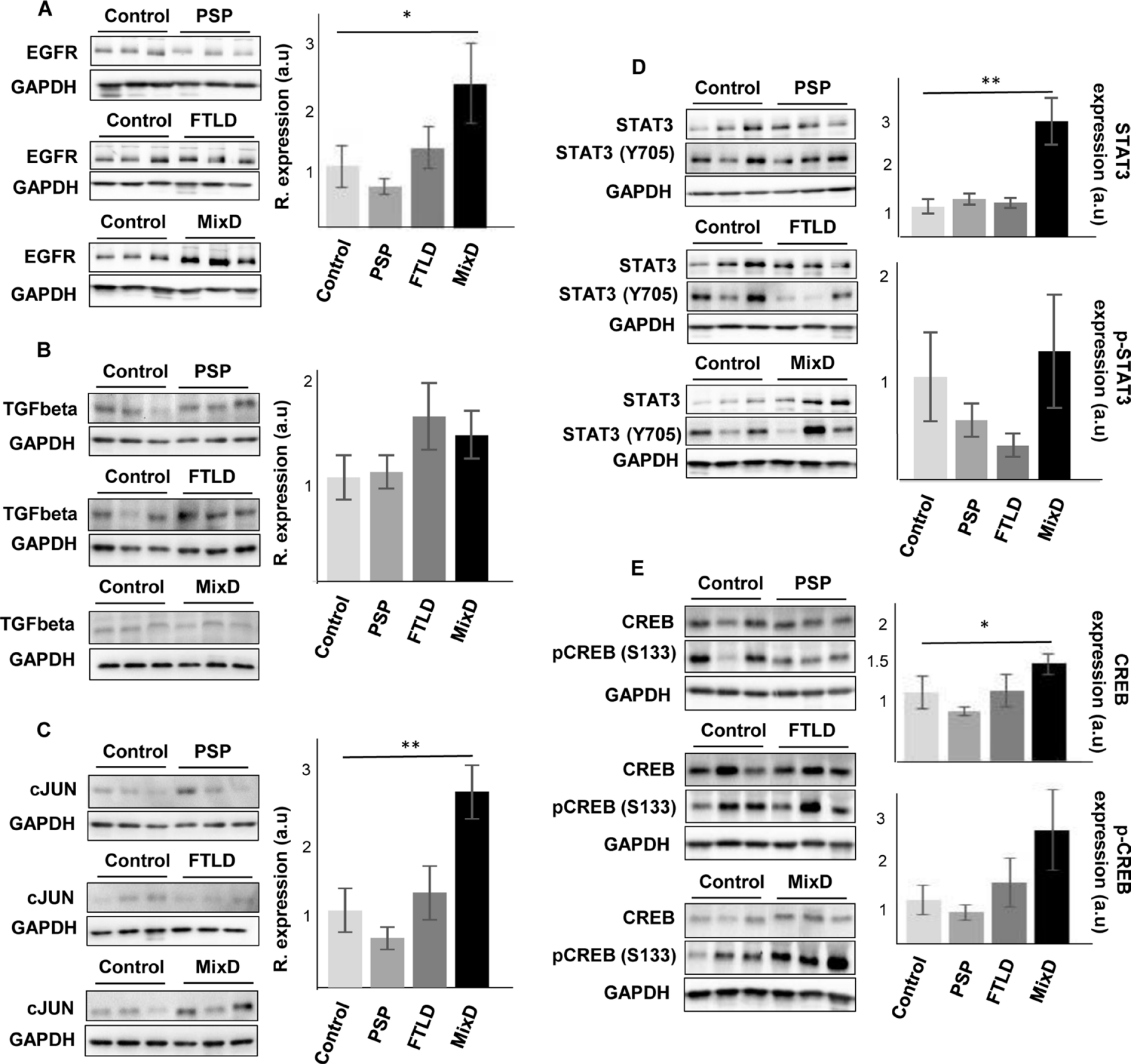
Olfactory bulb protein expression of EGFR, CREB1, TGF-beta, c-Jun and STAT3 across proteinopathies OB Protein expression was documented by Western blot. (**A**) EGFR expression, (**B**) TGF-beta expression, (**C**) c-Jun expression, (**D**) STAT3/phospho-STAT3 (Y705) expression, and (**E**) CREB/phospho-CREB (S133) expression, in PSP, FTLD, and mixed dementia subjects. Graphs represent histograms of band densities. Data are presented as mean ± SEM from: Controls (*n* = 4 cases), PSP (*n* = 9 cases), FTLD (*n* = 6 cases), and mixed dementia (mix AD VD) (*n* = 9 cases). *P < 0.05 vs control group; **P < 0.01 vs control group.

In contrast to the common separate investigation of neurological diseases, targeted cross-disease studies comparing shared molecular relationships may give new insights into possible olfactory perturbations common for all or some neurological disorders. In order to detect novel molecular features shared by different Alzheimer-related co-pathologies at olfactory level, we have evaluated the OB protein expression of EGFR, CREB1, TGF-beta, c-Jun, and STAT3 across several AD-related diseases (*n* = 28 OB samples). We have included pathologies with common smell impairment like FTLD [55, 56], PSP where olfactory loss occurs to a lesser extent or is absent [2, 57, 58], and mixed dementia. Mixed dementia is a condition in which AD and vascular dementia occur at the same time, and both separate disorders often display olfactory dysfunction [59, 60]. As shown in Figure 9, EGFR protein levels were also increased in the OB derived from mixed dementia subjects (Figure 9A). As previously observed in AD, OB TGF-beta levels were unchanged across PSP, FTLD, and mixed dementia (Figure 9B). In contrast, OB protein levels of STAT3 and CREB1 were significantly increased only in mixed dementia, without apparent shifts in their activation status (Figure 9D and 9E). Differently from AD, olfactory c-Jun protein levels were exclusively increased in mixed dementia (Figure 9C). c-Jun up-regulation has also been reported in entorhinal cortex and hippocampus from AD subjects and also in AD transgenic mice [61–63]. Mechanistically, the c-Jun N terminal kinase (JNK)/c-Jun cascade exerts its influence in aberrant processes of AD pathogenesis such as Tau hyperphosphorylation, amyloid aggregation, and synaptic dysfunction in murine models of AD [64–66]. According to previous studies [67, 68], the c-Jun overexpression observed in the OB of mixed dementia subjects could contribute to some AD-related neuropathologies present in vascular dementia such as beta amyloid-induced neuroinflammation and vascular insufficiencies.

## MATERIALS AND METHODS

### Materials

The following antibodies and materials were used: anti-GAPDH (Calbiochem), anti-EGFR (Millipore), anti-CREB, anti-phospho CREB (S133), anti-STAT3, anti-phospho STAT3 (Y705), anti-c-Jun (Cell signaling), and anti-TGF-beta (Abcam). Electrophoresis reagents were purchased from Biorad.

### Human samples

According to the Spanish Law 14/2007 of Biomedical Research, inform written consent form of the Neurological Tissue Bank of Navarra Health Service was obtained for research purposes from relatives of patients included in this study. The study was conducted in accordance with the Declaration of Helsinki and all assessments, post-mortem evaluations, and procedures were previously approved by the Clinical Ethics Committee of Navarra Health Service. Fourteen AD cases were distributed into different groups according to specific consensus diagnostic criteria [69–71]: low, intermediate, and high AD neuropathological changes (*n* = 4–5/group). Four cases from elderly subjects with no history or histological findings of any neurological disease were used as a control group. All human brains considered in this study had a post-mortem interval (PMI) lower than 10 hours (Table 1). Brain processing and the neuropathological study for protein deposits aggregates beta-amyloid and phospho-Tau were performed as previously described [34]. For the discovery phase, neuropathological assessment was performed according to standardized neuropathological scoring/grading systems, including Thal phases of beta-amyloid deposition, Braak staging of neuro fibrillary lesions, Consortium to Established a Registry for Alzheimer’s Disease, National Institute on Aging-Alzheimer’s Association (NIA-AA) guidelines, and primary age-related tauopathy (PART) criteria [69–73]. For the cross-disease analysis, different clinical backgrounds were considered: Progressive supranuclear palsy (PSP) (*n* = 9 cases; 4F/5M; median age: 74 years), frontotemporal lobar degeneration (FTLD) (*n* = 6; 3F/3M; median age: 81 years), mixed dementia (mix AD VD) (*n* = 9 cases; 4F/5M; median age: 85 years), and additional controls (*n* = 4; 1F/3M; median age: 80 years). In these cases, neuropathological assessment was performed according to standardized neuropathological guidelines: Mackenzie criteria for FTLD pathology [74], NINDS-AIREN criteria for vascular dementia [75], and NINDS criteria for PSP [76]. 80% of the OB samples included in the cross-disease phase had a PMI lower than 10 hours (Supplementary Table 1).

### Microarray hybridization and data analysis

For OB mRNA extraction, the Maxwell^®^ 16 simplyRNA Kit (Promega) was used. The sense cDNA was prepared from 1 ng of total RNA and then fragmented and biotinylated using Affymetrix GeneChip^®^ WT Pico Kit (PN902623). Labeled sense cDNA was hybridized to the Affymetrix Human Gene 2.0 ST chip according to the manufacturer protocols and using GeneChip^®^ Hybridization, Wash and Stain Kit. Genechips were scanned with the Affymetrix GeneChip^®^ Scanner 3000. For microarray data analysis, both background correction and normalization were done using RMA (Robust Multichip Average) algorithm [77]. Then, a filtering process was performed to eliminate low expression probe sets. Applying the criterion of an expression value of 16 in at least 2 samples for each experimental condition, 28353 probe sets were selected. R/Bioconductor was used for preprocessing and statistical analysis. For analysis of genes related to pathological changes, individuals with AD pathology were compared to non-demented controls. LIMMA (Linear Models for Microarray Data) was used to find out the probe sets that showed significant differential expression between controls and AD stages. We first used a threshold criteria of False Discovery Rate (FDR) < 5% to select differentially expressed genes. As in other transcriptomic studies performed in AD brains [15, 78], we did not achieve significant results using this criteria, so we worked with a *p*-value < 0.01 (without using any method for multiple testing correction). Microarray data files were submitted to the GEO (Gene Expression Omnibus) database and are available under accession number GSE93885.

The differential expression of RNAs was functionally analyzed through the use of Reactome [33], and QIAGEN’s Ingenuity^®^ Pathway Analysis (IPA) (QIAGEN Redwood City, www.qiagen.com/ingenuity), in order to detect and infer differentially activated/deactivated pathways as a result of AD phenotypes. IPA software comprises curated information from databases of experimental and predictive origin, enabling discovery of highly represented functions, pathways, and interactome networks.

### Western blotting

Equal amounts of protein (10 μg) were resolved in 12.5% SDS-PAGE gels. OB proteins derived from human samples were electrophoretically transferred onto nitrocellulose membranes for 45 min at 120 V. Equal loading of the gels was assessed by Ponceau staining. Membranes were probed with primary antibodies at 1:1000 dilution in 5% nonfat milk or BSA. After incubation with the appropriate horseradish peroxidase-conjugated secondary antibody (1:5000), antibody binding was detected by a ChemidocäMP Imaging System (Bio-Rad) after incubation with an enhanced chemiluminescence substrate (Perkin Elmer). All Band intensities were measured with Image Lab Software Version 5.2 (Bio-Rad) and normalized to GAPDH.

## CONCLUSIONS

Summing up, we have performed a stage-dependent comprehensive analysis of differential expression of OB coding transcripts during AD progression. To the best of our knowledge, this is the first study to characterize in depth, potential AD-associated transcriptional changes in the human OB. We performed gene set enrichment analysis to find the most relevant pathways and gene regulatory networks that are progressively modulated during AD progression. More importantly, using a discovery platform combining neuropathological diagnosis, OB transcriptome exploration, functional interaction data, together with a cross-disease analysis, a divergent olfactory expression of specific signal transducers has been observed across AD-related co-pathologies, serving as a foundation for new research areas into the role of olfactory signaling across different types of dementias.

## SUPPLEMENTARY MATERIALS FIGURES AND TABLES











## Abbreviations

AD: Alzheimer´s disease
CREB1: cAMP Responsive Element Binding Protein 1
c-Jun: AP-1 Transcription Factor Subunit
EGFR: Epidermal growth factor receptor
FTLD: Frontotemporal lobar degeneration
OB: olfactory bulb
PSP: Progressive supranuclear palsy
STAT3: Signal transducer and activator of transcription 3
TGF-beta: Transforming growth factor beta
