# Supplementary material for: Progressive modulation of the human olfactory bulb transcriptome during Alzheimer´s disease evolution: novel insights into the olfactory signaling across proteinopathies
Source: Oncotarget. 2017 May 23;8(41):69663–79. doi: 10.18632/oncotarget.18193 (PMC5642507; doi:10.18632/oncotarget.18193)
Supplement: Supplementary file 1 [file oncotarget-08-69663-s001.pdf]

Progressive modulation of the human olfactory bulb transcriptome during Alzheimer´s disease evolution: novel insights into the olfactory signaling across proteinopathies

SUPPLEMENTARY MATERIALS

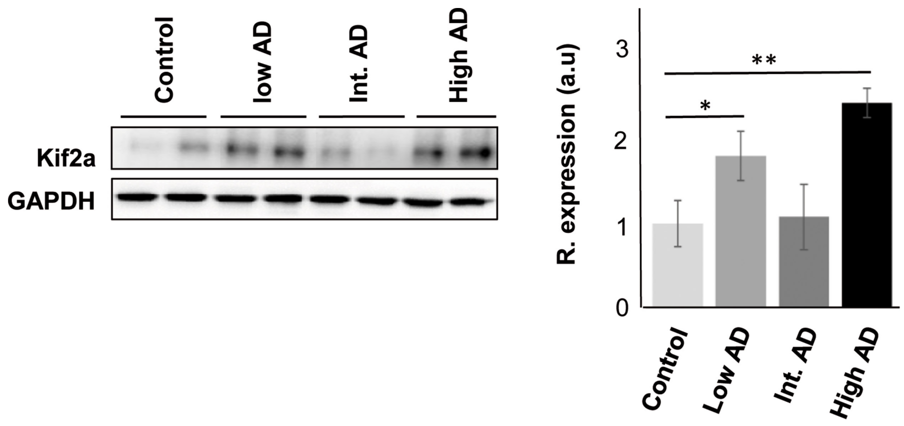

Supplementary Figure 1: Kif2a protein expression levels in the OB across AD staging.

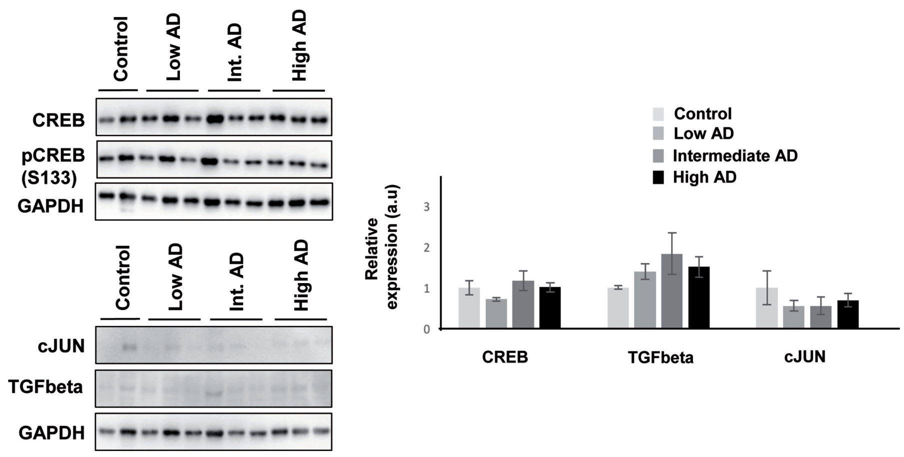

Supplementary Figure 2: Unmodified OB protein levels of CREB/pCREB, c-Jun, and TGF-beta across AD stages.

Supplementary Table 1: Subjects included in the cross-disease study

GROUPS	CASE (code)	AGE (years)	SEX	PMI	DEFINITIVE DX
Controls	C.1	88	F	3 h 30 min	PART (Braak II)+ Vascular disease
	C.2	79	M	11 h 25 min	PART (Braak I)+ Vascular disease
	C.3	62	M	8 h	PART (Braak II)
	C.4	91	M	4 h	PART (Braak II)
PSP	PSP.1	88	F	5 h	PSP+CAA+Thal1
	PSP.2	81	M	6 h 05 min	PSP +CAA+Thal 1
	PSP.3	80	M	3 h	PSP + Thal 4
	PSP.4	71	F	4 h	PSP
	PSP.5	70	M	3 h 30 min	PSP
	PSP.6	67	F	16 h	PSP
	PSP.7	78	M	16 h	PSP+AD A2B3C3
	PSP.8	85	F	2 h 30min	PSP +AD A1B2C2+CAA
	PSP.9	49	M	6 h	PSP
FTLD	FTLD.1	80	M	3 h 30 min	FTD-U
	FTLD.2	85	M	15 h	FTD-MND (TDP 43Type B)+ A2B1C1
	FTLD.3	78	F	2 h 20 min	FTD-TAU +(Pick disease)
	FTLD.4	77	F	3 h 50 min	FTD-TAU+(CDB)
	FTLD.5	78	M	4 h 30 min	FTD TDP 43 (Type B) +PART
	FTLD.6	89	F	5 h	FTD-TDP43(Type B)+A1B1C1
MIX dementia (AD VD)	MIXD.1	84	F	13 h	VD+AD (A1B1C3+Infarct in strategic areas)
	MIXD.2	84	F	2 h	VD+AD (A1B1C1+Multiple infarcts encephalopathy)
	MIXD.3	86	M	6 h	VD+AD (A1B1C3+Infarct in strategic areas)
	MIXD.4	79	M	8 h	VD+AD (A2B2C2 +Multiple infarcts encephalopathy)
	MIXD.5	84	M	3 h 25 min	VD+AD (A1B3C3+Infarct in strategic areas)
	MIXD.6	84	F	7 h 10 min	VD+AD (A1B2C3 + Multiple infarcts encephalopathy + Infarct in strategic areas)
	MIXD.7	87	M	12 h	VD+AD (A1B2C2 + Multiple infarcts encephalopathy)
	MIXD.8	88	F	14 h	VD+AD (A3B3C3 + Multiple infarcts encephalopathy)
	MIXD.9	82	M	2 h 50 min	VD+AD (A2B3C3 + Infarct in strategic areas)

Supplementary Table 2: Differential expressed OB protein-coding genes across AD grading. See Supplementary_Table 2

Supplementary Table 3: Meta-analysis based on previous transcriptomic experiments performed in hippocampal and cortical structures derived from AD patients. See Supplementary_Table 3

Supplementary Table 4: Potential interactome for human APP (β-amyloid precursor protein) and Tau protein using data mining-based methods for proteome-scale PPIs predictions (FpClass tool) [28]. See Supplementary_Table 4

Supplementary Table 5: Pathway analysis by Reactome tool. See Supplementary_Table 5
